# Double-contrast, single-phase computed tomography angiography for ruling out left atrial appendage thrombus prior to atrial fibrillation ablation

**DOI:** 10.1007/s10554-016-0973-2

**Published:** 2016-09-06

**Authors:** Cas Teunissen, Jesse Habets, Birgitta K. Velthuis, Maarten J. Cramer, Peter Loh

**Affiliations:** 1 Department of Cardiology, University Medical Center Utrecht, Heidelberglaan 100, 3584CX Utrecht, The Netherlands; 2 Department of Radiology, University Medical Center Utrecht, Utrecht, The Netherlands

**Keywords:** Atrial fibrillation, Catheter ablation, Computed tomography angiography, Left atrial appendage thrombus

## Abstract

Prior to atrial fibrillation (AF) ablation, computed tomography angiography (CTA) is increasingly used for left atrial appendage (LAA) thrombus detection. LAA filling defects on CTA may represent thrombus or incomplete contrast mixing with blood. A pre-bolus of contrast material with delay before the CTA contrast bolus can help distinguish between thrombus and incomplete contrast mixing. We present results from a double-contrast, single-phase CTA protocol used in our daily clinical practice. In patients who underwent AF ablation between 2011 and 2015, double-contrast, single-phase CTA was performed prior to ablation. Two contrast boluses (30 and 70 ml) with 25-s interbolus delay were administered followed by prospectively triggered cardiac CTA. Only patients with left atrial (LA) or LAA filling defects underwent transesophageal echocardiography (TEE) to rule out thrombus. Prior to ablation, 605 CTA-scans were performed (median radiation dose: 3.1 mSv). In 579 CTA-scans (95.7 %), the LA and LAA completely filled with contrast. In 26 CTA-scans (4.3 %) the LAA showed a filling defect whereby thrombus could not be excluded. In 2 of those 26 patients (7.7 % and 0.3 % of the total population), TEE verified LAA thrombus. Low-risk LAA filling defects on CTA (n = 7/26) with an inhomogeneous aspect, Houndsfield Unit values >100, and an indefinite border were all caused by incomplete contrast mixing. No thromboembolic complications occurred perioperatively or during 6 months follow-up. Prior to AF ablation, incidence of LAA filling defects on double-contrast, single-phase CTA is low. TEE remains warranted in all but low-risk filling defects to rule out thrombus.

## Introduction

Catheter ablation (CA) is a frequently performed treatment in patients suffering from drug-refractory atrial fibrillation (AF). Prior to CA, exclusion of left atrial (LA) and left atrial appendage (LAA) thrombus is recommended as its presence is a contraindication for CA [1]. Although transesophageal echocardiography (TEE) is considered the gold standard for excluding LA and LAA thrombus [1, 2], it is a semi-invasive procedure and associated with patient discomfort. Moreover, esophageal perforation is a very infrequent but potentially life-threatening complication [3–6]. Consequently, different modalities, like computed tomography angiography (CTA), are increasingly used as a screening tool for LA/LAA thrombi.

On CTA, LAA filling defects may represent thrombus but also incomplete mixing of contrast and blood due to slow flow. In order to distinguish better between thrombus and slow flow, different delayed CTA imaging protocols have been developed. Previous studies showed 99 % specificity of a second delayed-phase CTA [7, 8]. However, the extra radiation exposure of a delayed scan remains a concern. Therefore, in our daily clinical practice we use a single-phase, double-contrast injection protocol, with a delay between the first and second bolus. A pre-contrast bolus with delay before the acquisition contrast injection may increase the diagnostic accuracy by better enhancement of the LAA.

In this study, we present results from a large cohort of patients from an academic hospital setting who underwent double-contrast, single-phase CTA prior to AF ablation. All patients with LA or LAA filling defects on CTA underwent TEE. Goals of this study are to (1) demonstrate the incidence of LAA filling defects on CTA in daily clinical practice, (2) show the positive predictive value for LAA thrombus detection of this approach and (3) analyze LAA filling defect characteristics, representative for slow flow or thrombus.

## Methods

### Patient population

Our Institutional Review Board approved this study, and based on the retrospective observational nature of this study waived the requirement to obtain written informed consent. All consecutive patients suffering from symptomatic, drug-refractory or drug-intolerant AF who underwent CTA prior to primary or redo catheter ablation in the University Medical Center Utrecht from June 2011 to May 2015 were included. Transesophageal echocardiography was performed to verify or rule out thrombus in all patients with any type of LA or LAA filling defect on CTA. Computed tomography angiography and TEE were reviewed at the time of performance. In 2015, CTA and TEE were retrospectively re-evaluated by a cardiovascular radiologist and a cardiologist, blinded for outcomes.

Patient characteristics were prospectively collected and comprised sex, age, AF type, history of AF (years since AF was diagnosed), cardiovascular risk factors, CHA_2_DS_2_-VASc score, structural heart disease, LA size (end-systolic LA diameter in the parasternal long axis view on echocardiography) and anticoagulation therapy. Atrial fibrillation type was classified as paroxysmal (PAF), persistent or longstanding persistent according to the HRS/EHRA/ECAS 2012 Consensus Statement on Catheter and Surgical Ablation of AF [1].

### Cardiac computed tomography angiography

In all patients, double-contrast, single-phase, prospectively triggered cardiac CTA was performed ≤7 days prior to the ablation procedure to assess both LA and pulmonary vein (PV) anatomy, and exclude LA/LAA thrombus.

Computed tomography angiography was performed using a 256-slice CT system (Philips Healthcare, Cleveland, Ohio, USA). A 30 ml contrast bolus [Ultravist (iopromide) −300 mg I/ml, Bayer Schering Pharma AG, Berlin, Germany] was administered followed by a delayed 70 ml contrast bolus, 25 s after the first administration of contrast, both at a flow rate of 6 ml/s. After the second contrast bolus, a 60 ml saline chaser (30 % contrast and 70 % saline) was administered, followed by 30 ml of saline. Contrast was adjusted to patient’s body weight (>80 kg: 35 and 80 ml, respectively). The CTA-scan was initiated after the second contrast bolus injection using a monitoring scan and a predefined threshold of 200 Hounsfield Units (HU) in the ascending aorta with a post-threshold delay of 7 s to give breathing instructions. The scan parameters were as follows: collimation 128 × 0.625 mm, tube voltage 80–120 kV and tube current 195–210 mAs depending on weight (<65 kg, 65–80 kg, >80 kg), and rotation time 0.27 s. Images were reconstructed with a slice thickness of 0.9 mm and a reconstruction increment of 0.45 mm.

A filling defect was defined as an area of low attenuation in the LA or LAA, not caused by motion artifacts or other cardiac structures like atrial trabeculae. Houndsfield Units (HU) were measured throughout the area of low attenuation. Filling defects were classified as low, intermediate and high risk of thrombus based on the homogeneity of the region with low attenuation, HU-value and the border aspect. High risk filling defects consisted of homogeneous filling defects with low HU-values (<100 HU) [9] and a well-defined border (concave or convex). Low risk defects comprised inhomogeneous filling defects with high HU-values (>100 HU) and an ill-defined border. Filling defects were classified as intermediate risk of thrombus when both high and low risk characteristics were present.

Dose length products were registered during every examination. Radiation dose was calculated by the effective radiation dose: (dose-length product) × (conversion factor). The conversion factor varies for different body parts and for the heart it was set on 0.014 mSv/(mGy cm) [10].

### Transesophageal echocardiography

In case of a LA or LAA filling defect, TEE (iE33, Philips Healthcare, Best, The Netherlands) was performed after a median of five (4–6) days after CTA. State of the art 5-MHz multiplane probes were used to visualize LA and LAA thrombi. Within the LAA, low-flow states were analyzed by pulsed-wave Doppler velocity interrogation and by identification of spontaneous echocardiographic contrast (SEC). Spontaneous echocardiographic contrast was characterized by dynamic echogenic swirling patterns of blood flow.

### Anticoagulation strategy

Prior to CA, at least 4 weeks of adequate systemic anticoagulation with vitamin K antagonists (VKA) or novel oral anticoagulants (NOAC) was required in all patients irrespectively of the CHA_2_DS_2_-VASc score. Patients with a CHA_2_DS_2_-VASc score ≥2 were preferably treated with VKA. In VKA, target values for the international normalized ratio were 2.5–3.5. Vitamin K antagonists were continued peri-procedural. NOAC therapy was interrupted the day before CA and continued the day after.

During the ablation procedure, an intravenous bolus of heparin (≥5000 IU) was administered after trans-septal puncture, followed by additional boluses to maintain an activated clotting time between 300 and 350 s. After the ablation procedure, anticoagulation therapy was continued for at least 6 months. After 6 months, anticoagulation therapy was again individualized according to rhythm status and the CHA_2_DS_2_-VASc score.

### Assessment of thromboembolic complications

After the ablation procedure, patients remained routinely hospitalized for one day. Patients were seen on the outpatient clinic at 3 and 6 months after ablation. During hospitalization and at the outpatient clinic, occurrence of stroke and transient ischemic attack (TIA) was assessed. Stroke and TIA were defined according to the consensus report from the valve academic research consortium [11]. In short, diagnostic criteria for TIA comprised new focal neurological deficit with symptoms resolution within 24 h, without recent tissue injury on neuroimaging. Stroke was diagnosed if rapid onset of focal or global neurological deficit occurred, with a duration ≥24 h, and confirmed by a neurologist or neuroimaging [11].

### Statistical analysis

Categorical patient variables were shown as number and percentages. Continuous variables were expressed as means ± standard deviation or as median (25th–75th percentile), depending on the normality of the data distribution. For the comparison of continuous variables, an independent *t* test or a Mann–Whitney *U* test was performed. Differences in categorical variables between groups were compared by the Chi square test. Positive predictive value (PPV) of CTA was calculated, using TEE as the gold standard. A p value <0.05 was considered statistical significant. Statistical analyses were performed using SPSS 21.0 (IBM, Armonk, NY, USA).

## Results

**Fig. 1:**
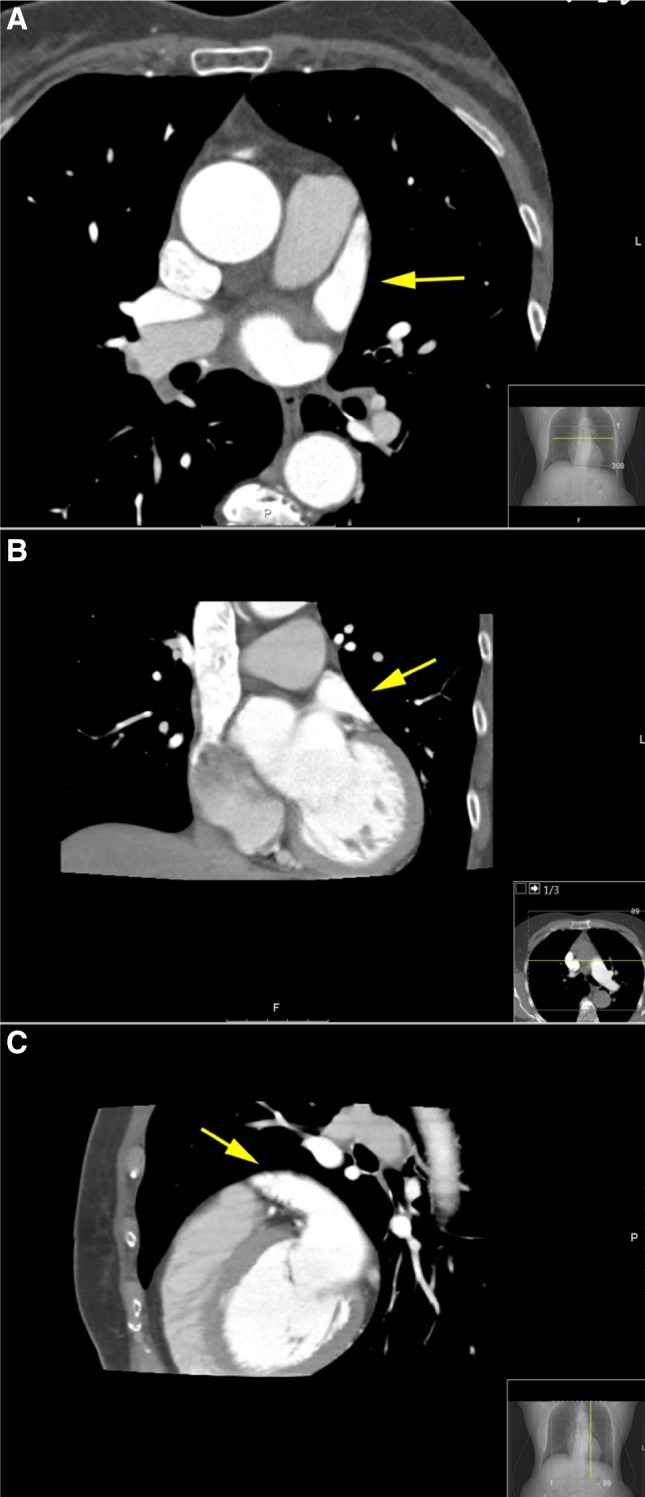
CTA showing complete filling of the LAA (*yellow arrow*), thereby excluding LAA thrombus. **a** Axial view, **b** coronal view, **c** sagittal view

From June 2011 till May 2015, 477 patients underwent 605 cardiac CTA scans prior to a total of 603 primary or redo CA procedures. No filling defects in the LA were seen in all 605 CTA scans. In 579 CTA scans (95.7 %), the LAA also completely filled with contrast (Fig. 1). In 26 CTA scans (4.3 %) the LAA showed a filling defect, whereby a thrombus could not be ruled out.

Patients characteristics, divided into presence and absence of LAA filling defects on CTA, are shown in Table 1. Patients with LAA filling defects were more often female (57.7 % vs. 23.9 %, p < 0.001), suffered more from (longstanding) persistent AF (65.4 % vs. 45.2 %, p = 0.011) and had a higher mean CHA_2_DS_2_-VAS_C_ score (2.4 ± 1.7 vs. 1.3 ± 1.3, p = 0.027).

**Table 1:** Baseline characteristics (n = 477)

Demographics	Negative CTA (n = 451)	Positive CTA (n = 26)
Male sex	343 (76.1 %)	11 (42.3 %)^†^
Age at first ablation (years)	59.2 ± 10.3	52.2 ± 7.6
AF type		
Paroxysmal	247 (54.8 %)	9 (34.6 %)^‡^
Persistent	178 (39.5 %)	12 (46.2 %)
Longstanding persistent	26 (5.8 %)	5 (19.2 %)
History of AF (years)	5.6 ± 5.6	6.2 ± 5.7
BMI (kg/m^2^)	27.1 ± 4.3	26 ± 4.1
Hypertension	174 (38.6 %)	8 (30.8 %)
Diabetes mellitus	37 (8.2 %)	4 (15.4 %)
CHA_2_DS_2_-VASc score	1.3 ± 1.3	2.4 ± 1.7^‡^
Atrial flutter	122 (27.1 %)	7 (26.9 %)
Structural heart disease	69 (15.1 %)	6 (23.1 %)
LA size (mm)	43 ± 7	46 ± 7
Anticoagulation		
VKA	350 (77.6 %)	22 (84.6 %)
NOAC	101 (22.4 %)	4 (15.4 %)

*Negative CTA* no LAA filling defect present on CTA, *Positive CTA* LAA filling defect present on CTA, *AF* atrial fibrillation, *BMI* body mass index, *CTA* computed tomography angiography, *LA* left atrium, *NOAC* novel oral anticoagulants, *VKA* vitamin K antagonist

^†^p value <0.01, ^‡^p value <0.05

Of the 26 patients with LAA filling defects on CTA, two showed LAA thrombus on TEE (PPV 7.7 %). In these two female patients (0.3 % of the total population), AF was continuously present prior to CTA for 3 and 10 months respectively. CHA_2_DS_2_-VAS_C_ scores were two and three, and in both patients INR was between 2.5 and 3.5. Catheter ablation was postponed in these two patients. After 3 months of optimal anticoagulation therapy, repeated TEE showed residual LAA thrombus in both patients and CA was cancelled.

In all 477 patients who underwent CA, no thromboembolic complications occurred perioperatively and during 6 months of follow-up. No major CTA related complications occurred (e.g. severe anaphylactic reaction to contrast or contrast-induced nephropathy). Five patients had a minor allergic reaction to contrast and all five made a full recovery. Median length product of all performed CTAs was 223 (138–230) mGy × cm, which corresponds to a median effective radiation dose of 3.1 (1.9–3.2) mSv.

### LAA filling defects on CTA

**Fig. 2:**
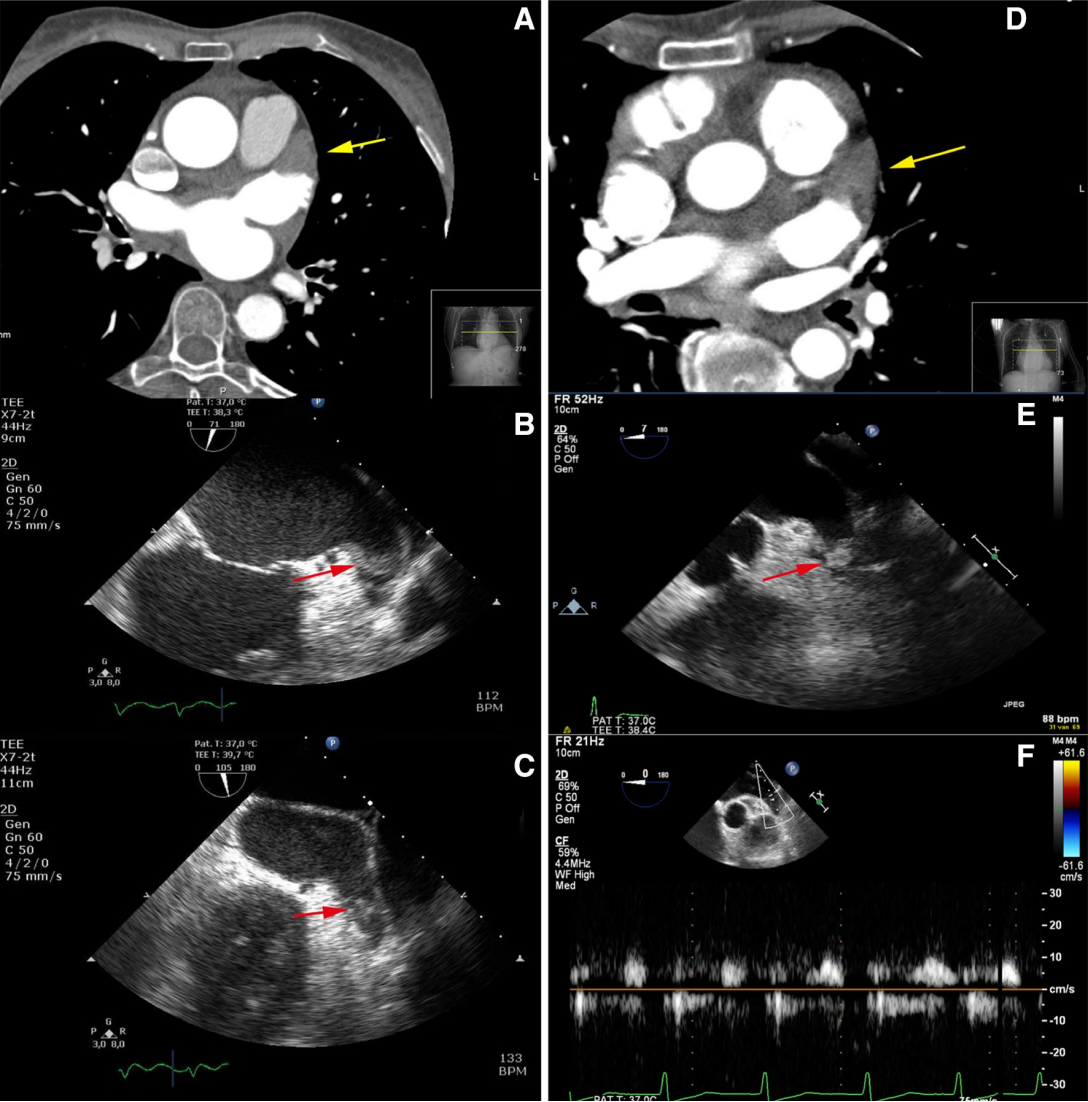
Two patients with a LAA filling defect on CTA based on thrombus. **a** CTA in axial view showing a LAA filling defect (*yellow arrow*). The area of low attenuation shows a homogeneous aspect, HU 50–70 and a well-defined concave border. **b, c** TEE showing a solid structure adherent to the LAA wall most likely thrombus (*red arrow*). **d** CTA in axial view showing LAA filling defect (*yellow arrow*). The area of low attenuation shows a homogeneous aspect, HU 70–90 and a well-defined convex border. **e** TEE showing LAA thrombus (*red arrow*). **f** Pulsed-wave Doppler showing low LAA flow velocity

**Fig. 3:**
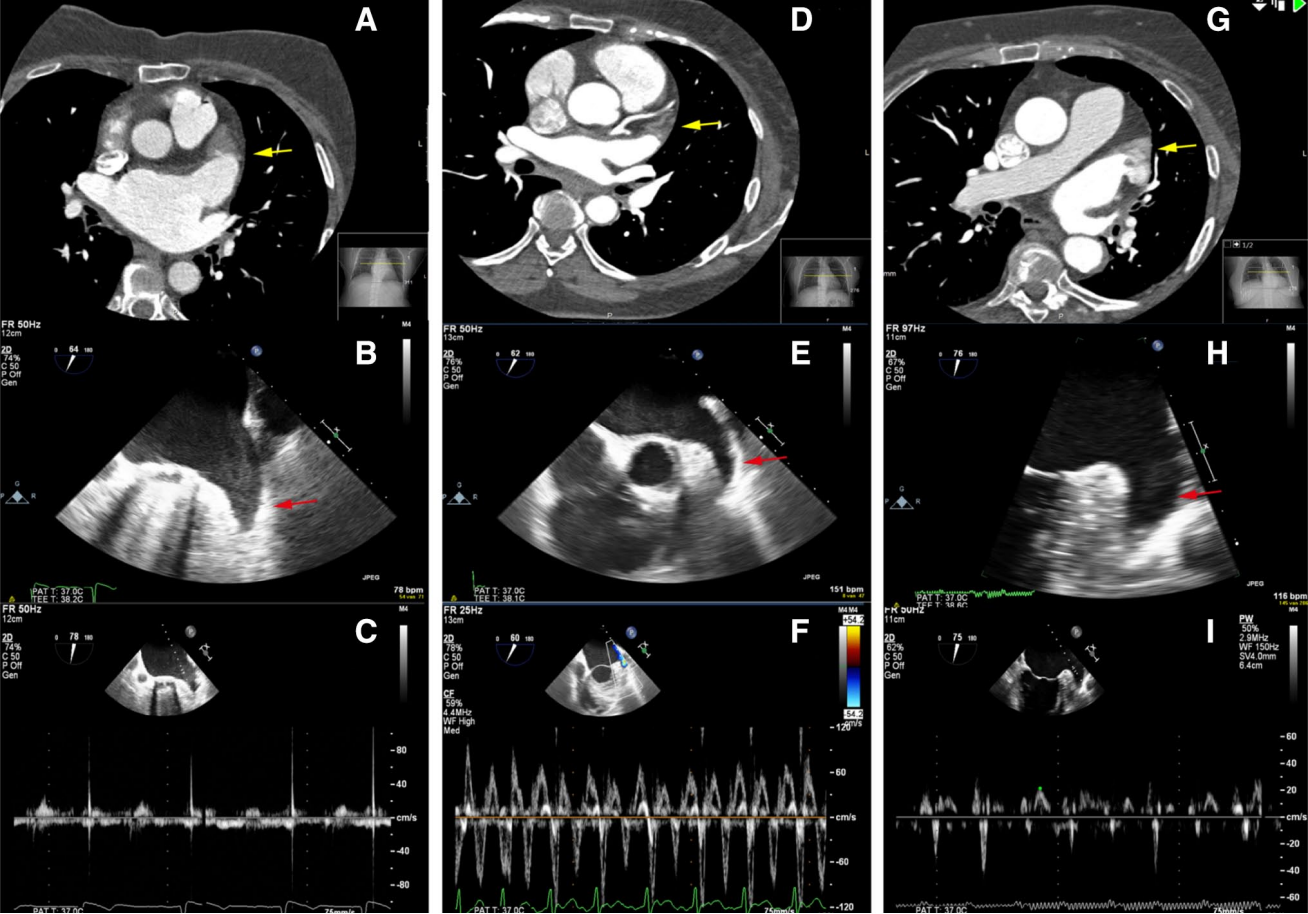
Three patients with false positive CTA scan. LAA filling defects were classified as high (**a**), intermediate (**d**) and low (**g**) risk of thrombus. The patients with a high and low risk filling defect were in AF during performance of CTA, the patient with an intermediate risk filling defect was in sinus rhythm. **a** CTA in axial view showing LAA filling defect (*yellow arrow*). The area of low attenuation shows a homogeneous aspect, HU 50–70 and a well-defined convex border. **b** TEE showing severe spontaneous echo contrast (*red arrow*) in the LAA without thrombus. **c** Low velocity measured by pulse Doppler. **d** CTA in axial view showing LAA filling defect (*yellow arrow*). The area of low attenuation shows a inhomogeneous aspect, HU 70–110 and a well-defined convex border. **e** TEE showing no thrombus and no spontaneous echo contrast (*red arrow*). **f** High velocity measured by pulse Doppler. **g** CTA in axial view showing LAA filling defect (*yellow arrow*). The area of low attenuation shows a inhomogeneous aspect, HU 150–250 and an ill-defined border. **h** TEE showing moderate spontaneous echo contrast in the LAA without thrombus (*red arrow*). **i** Low velocity measured by pulse Doppler

In 26 patients, LAA thrombus could not be ruled out by CTA. During acquisition of CTA, 17 of these 26 (65.4 %) patients were in AF. In 13 patients, the LAA filling defect had a homogeneous aspect with low HU values (<100) and a well-defined border. Those patients were classified as high risk of thrombus by an experienced cardiovascular radiologists. The remaining 13 patients were classified as intermediate (n = 6) and low risk (n = 7) of thrombus. Both patients with LAA thrombus on TEE had a high risk filling defect on CTA. Computed tomography angiography and TEE images of these two patients are shown in Fig. 2. No thrombi were found in the intermediate and low risk filling defect groups. Three examples of LAA filling defects (high, intermediate and low risk of thrombus) without thrombus on TEE are displayed in Fig. 3.

### LAA thrombus on TEE

Of the 26 patients with LAA filling defects on CTA, 2 patients showed LAA thrombus on TEE. Of the remaining 24 patients, 6 patients showed severe SEC, 8 patients moderate SEC and 8 patients no SEC. No TEE-associated complications occurred.

## Discussion

In this study we demonstrated that the incidence of LAA filling defects on double-contrast, single-phase CTA prior to AF ablation is very low. Yet, positive predictive value of CTA is low and TEE remains necessary to differentiate between thrombus and slow flow in case of LAA filling defects on CTA.

### CTA vs. TEE

Computed tomography angiography is a multifunctional modality. Besides exclusion of LAA thrombus, it is possible to create a detailed three dimensional visualization of the LA, LAA and PV anatomy. This anatomy can be merged into electroanatomical maps of the ablation procedure and may increase procedure efficacy. The use of only CTA, instead of both CTA and TEE, significantly reduces costs. Moreover, CTA is considered a patient friendly imaging modality, while TEE is a semi-invasive procedure with patient discomfort, and has infrequent, but potentially life threatening complications.

### CTA prior to AF ablation

The 2012 expert consensus statement on catheter ablation of AF states that CT imaging should not be used to screen for LA/LAA thrombi and that TEE is required. Prior to CA, TEE should be performed in all patients with AF more than 48 h in duration [1].This recommendation is based on two studies (n = 499) that showed reduced sensitivity (29 and 36 %) of CTA in LAA thrombi detection compared with TEE [12, 13]. Remarkably, a more recent meta-analysis, which included all 19 relevant studies (n = 2955), including the previously mentioned low sensitivity studies, showed an overall sensitivity of 96 %. Sixteen of the 19 included studies showed a sensitivity of 100 % [7]. Moreover, two studies using a similar double-contrast, single-phase CTA protocol showed 100 % sensitivity [14, 15]. This is congruent with our understanding that complete filling of the LAA with contrast excludes thrombus. Therefore, we considered it safe and ethical to perform TEE only in case of LA or LAA filling defects on CTA. This is supported by the fact that no strokes or TIAs occurred perioperatively and up to 6 months after ablation. Nevertheless, silent cerebral thromboembolic lesions could not be ruled out, since no cerebral magnetic resonance imaging (MRI) was performed prior to and after ablation.

Recently, a novel clinical protocol was presented for safely integrating results of CTA delayed imaging of the LAA into pre-procedure care [8]. Based on this protocol, TEE in addition to delayed-phase CTA was still necessary to exclude LAA thrombus in 24 % of patients. In contrast, using our protocol, TEE was only necessary in <5 % of patients.

### Different CTA imaging protocols

Lack of atrial contractions in AF cause low atrial filling and emptying velocities, as compared with individuals in sinus rhythm. As a result, contrast opacification of the LAA might take longer. Therefore, different delayed imaging protocols have been developed to reduce the number of false positive CTAs. Left atrial appendage thrombi detection can be performed by using single-phase and delayed-phase cardiac CTA. In single-phase CTA, images are obtained immediately after contrast administration. In delayed-phase CTA, images are obtained twice: immediately after contrast injection and with a 30–120 s delay after the contrast bolus. Earlier single-phase CTA studies showed a mean weighted sensitivity of 91 %, specificity of 95 %, PPV of 33 % and NPV of 100 % for LAA thrombus detection [7]. Recent studies using delayed-phase CTA demonstrated a sensitivity and NPV of 100 %, specificity of 99 % and PPVs ranging from 79 to 100 % [8, 16–19]. In case of LAA filling defects, delayed imaging seems more capable of differentiating between thrombus and slow flow [7]. However, a concern of delayed-phase imaging is the increased radiation dose of the double CTA scan.

Instead of a second delayed-phase CTA, a double-contrast, single-phase CTA scan (as used in our patients) can be performed by administering contrast twice with a delay between the first and second contrast bolus. The incidence of LAA filling defects of our approach was low (4.3 %). In comparison, mean incidence of LAA filling defects in 11 studies comparing CTA and TEE before atrial fibrillation ablation was 10.4 % [7]. Ten of the 11 studies were single-phase CTA, and none used a double-contrast injection. A potential disadvantage of a double-contrast protocol is the higher amount of contrast agent, slightly increasing the risk of contrast induced nephropathy.

Two recent studies compared double-contrast, single-phase CTA with TEE in patients with ischemic stroke. Both studies showed PPV of 100 % [14, 15]. Compared with our imaging protocol, a few major differences were noticed. First, the time between the first and second contrast bolus was 180 s, while we used a 25 s delay. Second, both studies used a 50 ml primary contrast bolus, while we used 30 ml of contrast. Last, in one study, patients underwent dual-energy CT [15]. In dual-energy CT, rapid kilovoltage switching allows simultaneous acquisition of low- and high-tube-voltage data sets. This makes it possible to measure iodine concentrations in LAA filling defects to differentiate between thrombus and slow flow. The study revealed that the mean iodine concentration was significantly lower in thrombus compared to slow flow. By using an iodine concentration cutoff value of 1.74 mg/ml, sensitivity and specificity were both 100 % for detection of LAA thrombus [15].

### Clinical implications

The presented CTA protocol could easily be adopted by other electrophysiology practices. It diminishes the need of TEE examinations and thereby increases patient comfort and reduces costs. Since low risk filling defects consist of benign characteristics (inhomogeneous aspect, HU-values >100 and an indefinite border), and no thrombi were detected in this group, not performing TEE in these patients might be considered. This would reduce the number of necessary TEEs to 3 %. However, due to the low event rate of true thrombus in our cohort, validation in other cohorts is recommended.

Based on previous studies, positive predictive value of CTA could be improved by increasing the amount of primary contrast bolus and prolonging the delay of the second contrast bolus. Performing a second delayed-phase CTA with extra radiation dose can be avoided in the majority of patients, but can still be considered if a filling defect is visualized on the first CTA scan. Finally, measuring iodine concentrations in dual-energy cardiac CT appears to be a promising technique to differentiate slow flow from LAA thrombus. Until recently the CT had to be programmed in advanced to acquire data sets with different kilovoltages. The clinical release of a new spectral detector-based CT (IQon, Philips Healthcare) can now provide this information retrospectively in all scans. More prospective studies are required to explore these novel acquisition techniques.

### Limitations

First, this was a retrospective cohort study with limitations associated with this study design. The low incidence of LAA filling defects and PPV in our study could only be compared with studies from other institutions.

Second, TEE was only performed in patients with LAA filling defects on CTA. Because of this approach, negative predictive value, sensitivity and specificity of CTA could not be calculated.

## Conclusion

Prior to AF ablation, incidence of LAA filling defects on double-contrast, single-phase CTA is low. TEE is still necessary to differentiate between thrombus and slow flow in case of LAA filling defects on CTA with high risk characteristics (homogeneous filling defects, low HU-values (<100 HU) or a well-defined border). In patients with low risk LAA filling defects, TEE might not be warranted.
